# Supplementary material for: Chemical features and machine learning assisted predictions of protein-ligand short hydrogen bonds
Source: Sci Rep. 2023 Aug 23;13:13741. doi: 10.1038/s41598-023-40614-7 (PMC10447522; doi:10.1038/s41598-023-40614-7)
Supplement: Supplementary file 1 — Supplementary Information. [file 41598_2023_40614_MOESM1_ESM.pdf]

# Supplementary Information

# Chemical Features and Machine Learning Assisted Predictions of Protein-Ligand Short Hydrogen Bonds

**Shengmin Zhou,[1,+] Yuanhao Liu,[2,+] Sijian Wang,[2,*] and Lu Wang[3,*]**

[1]YDS Pharmatech, Inc., Albany, NY 12226, USA; [2]Department of Statistics, Institute for Quantitative Biomedicine, Rutgers University, Piscataway, NJ 08854, USA; [3] Department of Chemistry and Chemical Biology, Institute for Quantitative Biomedicine, Rutgers University, Piscataway, NJ 08854, USA

[+]S.Z. and Y.L. contributed equally to this work.

[*]To whom correspondence should be addressed: sijian.wang@stat.rutgers.edu, lwang@chem.rutgers.edu and

## 1. Supplementary methods

### 1.1 Protein structure preparation and hydrogen bond analysis

From the PDB, we obtained 1070 structures of protein-ligand complexes that were refined from X-ray or neutron scattering experiments and had resolution greater than or equal to 1.1 Å. These structures were deposited in the database between 1994 and 2019, with 35.7% of them deposited after 2015. The following table provides the breakdown of their year distribution.

| Year | Number of structures | Year | Number of structures |
|---|---|---|---|
| 1994 | 2 | 2008 | 43 |
| 1995 | 1 | 2009 | 32 |
| 1997 | 1 | 2010 | 57 |
| 1998 | 6 | 2011 | 62 |
| 1999 | 4 | 2012 | 59 |
| 2000 | 8 | 2013 | 68 |
| 2001 | 18 | 2014 | 87 |
| 2002 | 29 | 2015 | 69 |
| 2003 | 36 | 2016 | 78 |
| 2004 | 43 | 2017 | 76 |
| 2005 | 51 | 2018 | 111 |
| 2006 | 42 | 2019 | 48 |
| 2007 | 39 | | |

99.5% of the 1070 protein-ligand complexes had R-factor $\leq 0.20$ and $R_{free} - R \leq 6\%$, demonstrating that the dataset contained high-quality crystal structures.[1] Analysis conducted using the CD-HIT program[2,3] revealed sequence redundancy among the proteins in the dataset. By applying a sequence identity cutoff of 0.9, we observed a reduction from 1113 chains to 502 independent chains. However, we retained all the structures for the hydrogen bond analysis because they contained distinct small molecule ligands despite sharing the same protein structures. We expect that the MAPSHB-Ligand model will effectively capture the structural features of protein-ligand SHBs regardless of potential redundancy in the protein sequences in the dataset. The PDB IDs of the protein-ligand complexes are listed in Table S9.

From the protein structures, we removed the crystallographic water molecules and analyzed the hydrogen bonds that formed between amino acids or between amino acids and small molecule ligands using the Amber 2016 package.[4] A pair A-H$\cdots$B was treated as a hydrogen bond if the heteroatoms were O or N atoms, 2.3 Å $\leq$ R $\leq$ 3.2 Å, and the A-H-B angle $\geq 135°$. It was then further categorized as a SHB if R $\leq$ 2.7 Å, and a NHB if R $\geq$ 2.8 Å. A distance interval of 0.1 Å was used to better distinguish the two classes of hydrogen bonds. We found that 6.5% of the amino acids involved in the SHBs were near the C- or N-terminus of a protein and did not have the neighboring 3 residues that were used as the sequence information in the model development. In addition, 0.4% of the amino acids were in structurally uncertain regions of proteins that showed multiple possible amino acids in one position. We removed all of these cases in the hydrogen bond analysis since they occurred only rarely in the dataset. Furthermore, we excluded inorganic anions and small inorganic molecules, such as $SO_4^{2-}$, $PO_4^{3-}$, and polyols such as ethylene glycol and glycerol in the analysis of protein-ligand SHBs. These ligand molecules and ions are mainly used in the solvation of biomolecules to prepare for the X-ray and neutron scattering experiments and are not likely to possess biological functions. The chemical IDs of the excluded ligands are listed in Table S4 and the resulting ligands involved in protein-ligand SHBs are provided in Tables S4 and S10.

The numbers of SHBs and NHBs observed in the high-resolution protein structures are summarized in Tables S1 and S2. When protein backbone is involved, the probability of observing protein-protein and protein-ligand SHBs are below 3% and 6%, respectively. As such, we only consider amino acid side chains for the hydrogen bond analysis in this work. We observe

7070 SHBs and 22353 NHBs that form between the side chains of amino acids and they are distributed in 1061 structures of protein-ligand complexes. When small molecule ligands are involved, we find a total of 1272 SHBs and 2733 NHBs that are distributed in 917 crystal structures.

### 1.2 Development and analysis of the machine learning model

To develop the MAPSHB-Ligand model, we randomly partitioned the overall dataset of protein-ligand hydrogen bonds with an 80:20 ratio. This yielded 1019 SHBs (31.7%) and 2200 NHBs (68.4%) in the training dataset, and 253 SHBs (32.2%) and 533 NHBs (67.8%) in the test dataset. We note that recent studies have highlighted the potential limitations of using a random split to form the training and testing datasets.[5] Specifically, new data may differ from the observations in the current dataset, so evaluating the model solely based on a random split may lead to an overestimation of its performance.[5] In the field of structural biology, there are continuous advancements that improve the effectiveness of protein structure determination and refinement and new techniques may introduce variations in future protein-ligand complexes. However, since we have focused on atomic-resolution protein structures in the PDB, which represent the highest quality structures available to date, it is unlikely that protein-ligand complexes determined in future studies will exhibit significantly different patterns compared to the existing data. To improve the treatment of data in this work, one potential approach is to use historical data for training and more recent data for evaluation. However, given the relatively small size of the dataset and the fact that only 35.7% of the structures are deposited after 2015, relying solely on historical data in the training of the machine learning model may result in the loss of crucial patterns in the data. As more high-resolution protein structures become available, we will continue to update and refine the model to ensure its effectiveness in capturing the evolving patterns and trends in protein-ligand SHBs.

Similar to the MAPSHB model,[6] the MAPSHB-Ligand model must tackle two challenges. First, SHBs only account for 31.7% of the hydrogen bonds in the training dataset. Based on this imbalanced dataset, a standard machine learning model is more likely to classify a given hydrogen bond as a NHB than a SHB. Second, the 14 input features display strong interaction effects when predicting the formation of SHBs. For example, the effect of ligand functional group is highly dependent on the type of amino acid residue present. As an example, Thr shows SHB probabilities of 39% and 14% when paired with the hydroxyl and amine groups of ligands, respectively. The odds ratio is $\left(\frac{39\%}{1-39\%}\right) / \left(\frac{14\%}{1-14\%}\right) = 3.9$. In comparison, Glu has probabilities of 85% and 11% for forming protein-ligand SHBs with the hydroxyl and amine groups of ligands, respectively. The odds ratio now becomes $\left(\frac{85\%}{1-85\%}\right) / \left(\frac{11\%}{1-11\%}\right) = 45.8$, which is 11 times larger than that of the previous case. Therefore, it is crucial for the model to explicitly account for the interaction effects among the input features when it predicts the class of a protein-ligand hydrogen bond.

As described in the main text, we chose the undersampling technique to overcome the class imbalance problem, and the gradient boosting models as blocks to handle the interaction effects of the input features in the construction of the MAPSHB-ligand model. In the following, we provide a detailed description of the gradient boosting model and discuss the evaluation metrics used to assess the model predictions. We then assess the necessity of using the undersampling strategy in the development of the machine learning model.

#### *1.2.1 Gradient boosting model*

Gradient boosting is a commonly used machine learning algorithm for classification tasks. It is an ensemble method that improves the prediction accuracy by combining the outputs of multiple weaker models, such as decision trees.[7] The main concept behind gradient boosting is to iteratively add new base learners to the model, with each one focusing on the errors made by the previous model and attempting to correct them. In other words, each subsequent model in the sequence primarily concentrates on the erroneous predictions made by the preceding models, which enables the algorithm to gradually correct and refine the predictions.

As shown in Algorithm 1,[8] the input dataset of each protein-ligand hydrogen is $\mathcal{D} = \{\mathbf{x}_i, y_i\}_{i=1}^{N}$, where $\mathbf{x}_i$ are the 14 input features of the $i^{th}$ hydrogen bond and $y_i$ is its class. $y_i$ takes the value of -1 for a NHB, and +1 for a SHB. The gradient boosting model initializes the model, denoted as $F_0(\mathbf{x})$, with a constant value that can minimize the exponential loss function. After initialization, the gradient boosting model takes $M$ steps to train $M$ decision trees and average them to obtain the final model. In each step, a decision tree is fitted to predict the negative gradients of the loss function with respect to the predictions of the model in the previous step. Figure S1 illustrates a fitted decision tree. The algorithm then updates the model by adding the newly fitted decision tree to the previous one. The output of the gradient boosting model is the predicted probability that a hydrogen bond belongs to a SHB.

As discussed in the main text, the MAPSHB-Ligand model predicts the SHB probability by averaging over the outputs of 10 gradient boosting models. The predicted probability is then compared with a pre-determined classification threshold to obtain the class of a hydrogen bond. If the probability is greater than or equal to the threshold, the hydrogen bond is classified as a SHB. If the probability is lower than the threshold, it is classified as a NHB.

**Algorithm 1** The gradient boosting method (adapted from Algorithm 1 of reference 8)

**Inputs:** $(\mathbf{x}_1, y_1) \cdots, (\mathbf{x}_N, y_N)$
**Exponential Loss Function:** $L(y, F) = e^{-yF}$
**Step 1:** Initialize the model $F_0(\mathbf{x}) = \arg\min_\rho \sum_{i=1}^{N} L(y_i, \rho)$
**Step 2:**
**for** $m = 1, \cdots, M$ **do**

1. Calculate the steepest-descent direction

$$\tilde{y}_i = -\left[\frac{\partial}{\partial F(\mathbf{x}_i)} L(y_i, F(\mathbf{x}_i))\right]_{F(\mathbf{x})=F_{m-1}(\mathbf{x})}, i = 1, \cdots, N$$

2. Fit a decision tree model using

$$\mathbf{a}_m = \arg\min_{\mathbf{a},\beta} \sum_{i=1}^{N} [\tilde{y}_i - \beta h(\mathbf{x}_i; \mathbf{a})]^2$$

Here $h(\mathbf{x}; \mathbf{a})$ is a decision tree with parameter $\mathbf{a}$.

3. Calculate the line search

$$\rho_m = \arg\min_{\rho} \sum_{i=1}^{N} L(y_i, F_{m-1}(\mathbf{x}_i) + \rho h(\mathbf{x}_i; \mathbf{a}_m))$$

4. Update the model

$$F_m(\mathbf{x}) = F_{m-1}(\mathbf{x}) + \rho_m h(\mathbf{x}; \mathbf{a}_m)$$

**Step 3:** Calculate the predicted probability that $\mathbf{x}$ belongs to the SHB class

$$p(\mathbf{x}) = \frac{1}{1 + e^{-2F_M(\mathbf{x})}}$$

#### *1.2.2 Evaluation metrics*

We consider the following four metrics to evaluate the MAPSHB-ligand model: precision, recall, the receiver operating characteristic (ROC) curve and the area under the curve (AUC). These metrics are defined based on a confusion matrix. As shown below, the confusion matrix has four entries: True Positive (TP), False Positive (FP), False Negative (FN), and True Negative (TN). TP represents the number of SHBs that are correctly classified as SHBs; FP represents the number of NHBs that are incorrectly classified as SHBs; FN represents the number of SHBs that are incorrectly classified as NHBs; TN represents the number of NHBs that are correctly classified as NHBs.

**Confusion Matrix**

| | Predicted SHB | Predicted NHB |
|---|---|---|
| Real SHB | True Positive (TP) | False Negative (FN) |
| Real NHB | False Positive (FP) | True Negative (TN) |

The evaluation metrics are defined as

$$\text{Recall} = \frac{\text{True Positive}}{\text{Real SHB}} = \frac{\text{TP}}{\text{TP} + \text{FN}}$$

$$\text{Precision} = \frac{\text{True Positive}}{\text{Predicted SHB}} = \frac{\text{TP}}{\text{TP} + \text{FP}}$$

$$\text{False Positive Rate} = \frac{\text{False Positive}}{\text{Real NHB}} = \frac{\text{FP}}{\text{FP} + \text{TN}}$$

Therefore, recall quantifies the amount of SHBs that are correctly identified by the model out of all the SHBs in the test dataset, and precision measures how many predicted SHBs are real SHBs. False positive rate (FPR) is the proportion of NHBs that are incorrectly identified as SHBs by the model. Among these evaluation metrics, larger values of recall and precision and smaller values of FPR indicate better model performance in distinguishing SHBs from NHBs.

We apply the MAPSHB-Ligand model to the test dataset and calculate the precision and recall of its predictions for different classification thresholds. From Figure 4a and Table S7, both metrics depend on the choice of the classification threshold and there is a trade-off between them. As a result, it is not feasible to find a threshold that maximizes precision and recall at the same time. To further evaluate the model, we plot an ROC curve and compute the AUC score as a metric that is

independent of the choice of the classification threshold. As demonstrated in Figure 4b, the ROC curve is obtained by varying the classification threshold from 0 to 1, and each point corresponds to a (FPR, recall) pair at a given classification threshold. If the threshold is set as 0, all hydrogen bonds would be predicted as SHBs and it gives a point of (FPR=100%, recall=100%) on the ROC curve. Conversely, all the hydrogen bonds would be classified as NHBs if a threshold value of 1 is used, leading to a point of (FPR=0, recall=0) on the curve.

A perfect classification model assigns higher probabilities to real SHBs than real NHBs and its ROC curve is a combination of two straight lines. It starts at the point of (FPR=0, recall=0) when the classification threshold is 1, and follows a vertical line to reach (FPR=0, recall=100%) as the threshold decreases. After all the SHBs have been identified, further reducing the threshold does not alter the recall rate but increases the FPR. The ROC curve hence follows a horizontal line at recall=100% until it reaches the point of (FPR=100%, recall=100%). The AUC score for such a perfect model is 1. In contrast, a classification model that gives completely incorrect predictions assigns higher probabilities to real NHBs than real SHBs and its ROC curve also consists of two lines. It moves horizontally from the point of (FPR=0, recall=0) to (FPR=100%, recall=0) when the classification threshold decreases from 1 to the value where all the NHBs are predicted as SHBs. As the threshold further decreases, the recall rate starts increasing while the FPR remains at 100%, so the ROC curve moves vertically to the point of (FPR=100%, recall=100%). The resulting AUC score is 0. Most classification models fall between the completely incorrect model and the perfect model and their AUC scores are between 0 and 1. For example, a model that classifies hydrogen bonds by randomly tossing a fair coin would predict a 50% probability for any hydrogen bond to be a SHB. If the classification threshold is set to a value below 0.5, all the hydrogen bonds would be predicted as SHBs, resulting in a point of (FPR=100%, recall=100%) in the ROC curve. If the threshold is instead set to a value greater than or equal to 0.5, all the hydrogen bonds would be predicted as NHBs, leading to a point of (FPR=0, recall=0). The ROC curve is then a diagonal line connecting these two points on the plot and the corresponding AUC is 0.5. Therefore, the ROC curve and the AUC score are useful tools to identify how well the MAPSHB-Ligand model distinguishes between SHBs and NHBs.

#### *1.2.3 Gradient boosting-only model*

Given that the number of NHBs is only twice that of SHBs in the training dataset, the class imbalance issue for the protein-ligand SHBs exists but is not significant. This indicates that applying the gradient boosting model directly, without the undersampling strategy, would likely yield similar results. We hence repeated the development of the machine learning model without the undersampling method and obtained a gradient boosting-only model.

As shown in Figure S2, the ROC curve of the gradient boosting-only model closely resembles that of the MAPSHB-Ligand model and their AUC scores are both 0.96. These metrics suggest that the two models have similar overall performance.

Next, we evaluated the precision and recall of the gradient boosting-only model at various classification thresholds. Comparing the results from Tables S7 and S8, we find that the gradient boosting-only model tends to make more conservative predictions, favoring the classification of a given hydrogen bond as an NHB. For example, when the threshold is set to 0.996, the model achieves a precision of 100% on the test dataset. However, this high precision comes at the expense of a low recall of only 38%, indicating that very few true SHBs are detected in this case. Similarly, at the recommended classification threshold of 0.870, the gradient boosting-only model shows a precision of 88%, but it only identifies 72% of SHBs in the test dataset. Since our main objective is to identify SHBs in protein-ligand complexes, and considering that these close contacts are relatively rare in real applications, we combine the undersampling strategy with the gradient boosting model in the development of the MAPSHB-Ligand model to enhance the discovery of SHBs.

## 2. Supplementary figures

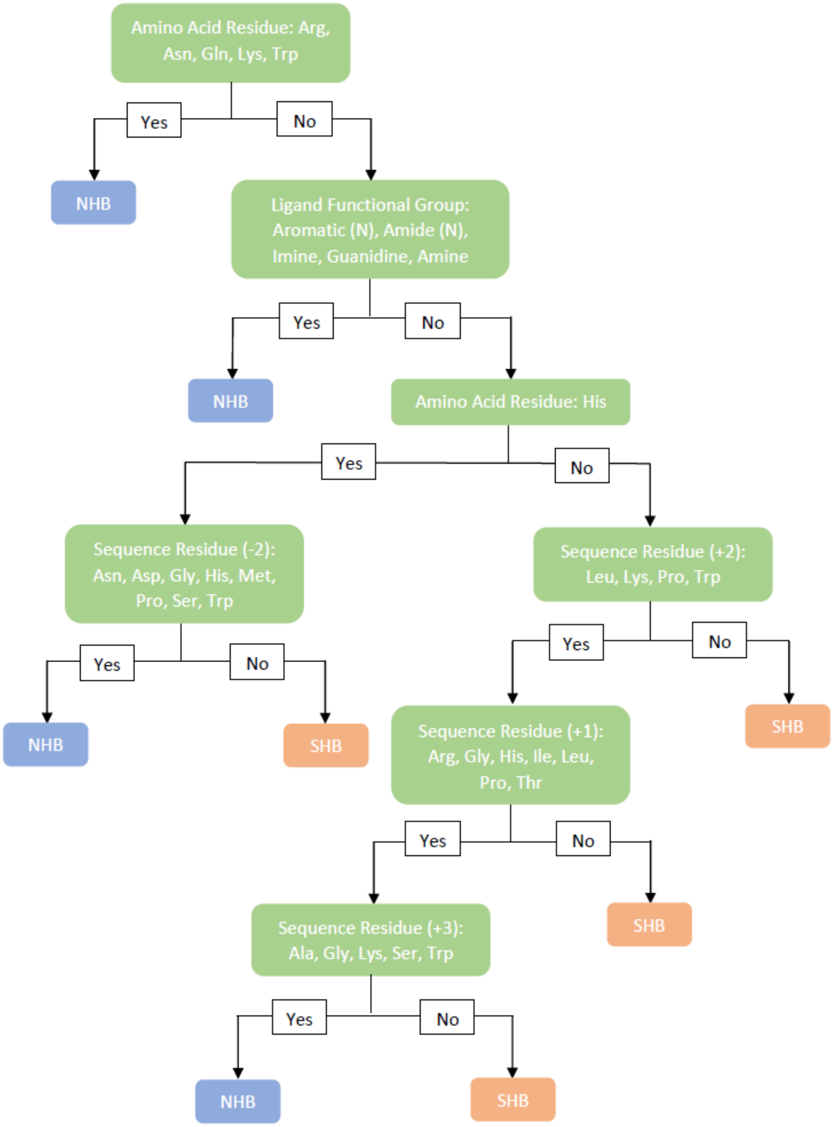


**Figure S1.** An example decision tree in the gradient boosting model. The green boxes represent the conditions of the judgements. The gradient boosting model fits $M$ decision trees for each balanced dataset and average over their predictions, as explained in Algorithm 1.

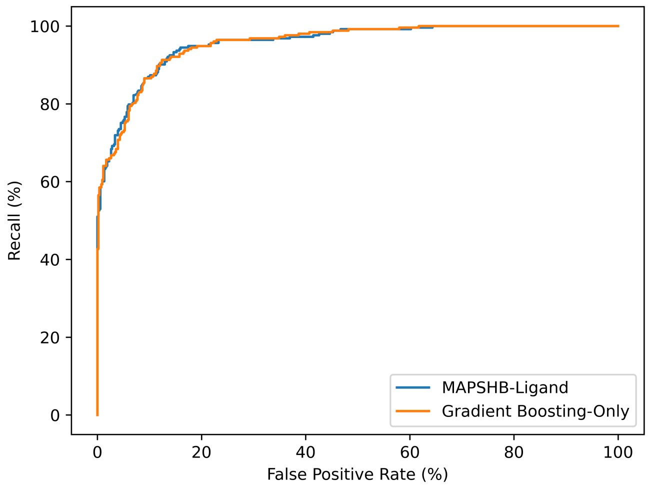


**Figure S2.** ROC curves of the MAPSHB-Ligand and gradient boosting-only models. The two ROC curves closely resemble each other.

## 3. Supplementary tables

**Table S1. Summary statistics of protein-protein hydrogen bonds when the amino acid residues are in the backbone or side chain of proteins. Given the low probability of observing a SHB when the protein backbone is involved, we only consider the side chain-side chain hydrogen bonds in the analyses.**

| Location of amino acids | Number of SHBs | Number of NHBs | Percentage of SHBs |
|---|---|---|---|
| Backbone-Backbone | 436 | 152921 | 0.3% |
| Backbone-Side chain | 3927 | 50314 | 7.2% |
| Side chain-Side chain | 7070 | 22353 | 24.0% |
| Total | 11433 | 225588 | 4.8% |

**Table S2. Summary statistics of protein-ligand hydrogen bonds when the amino acid residues are in the backbone or side chain of proteins. Given the low probability of observing a SHB when the protein backbone is involved, we only consider the hydrogen bonds formed between the side chains of amino acids and ligands in the analyses.**

| Location of amino acids | Number of SHBs | Number of NHBs | Percentage of SHBs |
|---|---|---|---|
| Backbone | 121 | 2230 | 5.1% |
| Side chain | 1272 | 2733 | 31.8% |
| Total | 1393 | 4963 | 21.9% |

**Table S3. Summary statistics of the types of heteroatoms in protein-ligand hydrogen bonds.**

| Heteroatoms (protein atom-ligand atom) | Number of SHBs | Number of NHBs | Percentage of SHBs |
|---|---|---|---|
| O-O | 1032 | 280 | 78.7% |
| O-N | 24 | 556 | 4.1% |
| N-O | 214 | 1788 | 10.7% |
| N-N | 2 | 109 | 1.8% |
| Total | 1272 | 2733 | 31.8% |

**Table S4. PDB chemical IDs for the small molecule ligands that are excluded in the analysis or are involved in protein-ligand SHBs and in the 5 main categories. A full list of ligands in the protein-ligand SHBs is provided in Table S10.**

| Ligand Category | Chemical IDs |
|---|---|
| Excluded | HOH WAT DOD OH NH3 NH4 CYN NO3 CO2 CO3 BCT SO4 PO4 PI WO4 8AR CAD SE4 PO3 NO2 NO POP EOH GOL EDO PEG MOH PGE PGO PG4 1PE NH2 OXY F CAC AZI DMS SCN P33 XPE HEZ IPA BU3 15P MXE DIO PE4 P6G ME2 ETF DXE |
| Carbohydrates | GLC BMA MAN BG6 ADA RAM CBI E4P F6R BGC NAG GAL LMT MMA FRU FUC NDG 5RP XYS GN1 XYP MGL MFU G7P MA4 AHR PRP LAT CE5 BOG GTR GLA IDC 4IP SGC SSG A2G |
| Nucleotides | FAD FMN FAE BYN NAP NAD NAI NAJ NDP ODP 8GD URI 6OG 7CI UDP 8DG AP5 B4P AMP ATP KBD UMC GDP ADP ACP AMZ G3D NEU 3AM U1P APC U5P GNP ACO JBT 5GP SAM DCM COA 6U4 MCN ADN |
| Acids or anions | FMT CIT SAL ACT FLC MHA OCA TLA SIN ACA HLP IAC FQZ 3DS XZA ZPO HHT HJK HGH HHB HHW SIA PLM DKA ACY AKG S3P TFA ZGB GRI ISC HCA NIO MLT TAR 01F 3EB BEZ 173 OXM FOL PYR DAO MLI 0V5 KI2 PPF EDT HC4 PHB FER MCO 1DF |
| Hemes | HEM HEC FDD FDE 6HE |
| Amino acids | DLY DTH DCY ALO OCS OGA GHP OMY AIB DLE DAS DSN DGL DAL DTR TPO CCS MEA 9KK B2H SAH DA2 PCA MSE HYP CSO OHI ACE GYS CRO PHL CSD CXM 23F VDL VLL D3P NLE LYZ TRQ KOR SNC CGU OSE M3L CSX ZZU BB9 SLB ETA FVA HM8 MLY SEP AGD C6L FME MDO CSS MIR DCL OLD IYR TPQ QIL C4L FP9 GSF GTS PIA DIV AGM MGN DYA MHS SMC MHO PTR MLZ HIC VOL SUI DBB |

**Table S5. Ligand functional groups and their abbreviations used in the MAPSHB-Ligand model. Ph represents the phenyl group and R represents an arbitrary functional group.**

| Ligand functional group | Abbreviation | Ligand functional group | Abbreviation |
|---|---|---|---|
| Aromatic (N) | NA | Carboxylate | OATE |
| Amide (N) | NAM | Ester (-R) | OET |
| Nitrile (-CN) | NC | Carboxyl (=O) | OIC |
| Imine | NIM | Carboxyl (-OH) | OICH |
| R-Ph-NH | NPHH | R-N-O | ON |
| Guanidine | NQUA | Hydroxyl | OOL |
| Amine | NRH | Ketone/Aldehyde | OONE |
| R-S-N- | NS | Phosphate | OP |
| Aromatic (O) | OA | Phenol | OPHH |
| Amide (O) | OAMI | Sulfate | OS |

**Table S6. Examples of commonly observed protein-ligand SHBs in the dataset. The location of the ligand is in the donor (D) or acceptor (A) of a SHB.**

| Amino acid | Ligand functional group | Ligand location | Number of SHBs | Number of NHBs | Percentage of SHBs |
|---|---|---|---|---|---|
| Asp | Hydroxyl | D | 328 | 47 | 87.5% |
| Glu | Hydroxyl | D | 202 | 35 | 85.2% |
| Ser | Phosphate | A | 37 | 28 | 56.9% |
| Thr | Phosphate | D | 29 | 0 | 100.0% |
| Asp | Carboxyl (-OH) | D | 28 | 12 | 70.0% |
| His | Carboxyl (=O) | A | 27 | 10 | 73.0% |
| Tyr | Hydroxyl | A | 24 | 6 | 80.0% |
| Tyr | Carboxyl (=O) | A | 24 | 2 | 92.3% |
| Thr | Phosphate | A | 21 | 2 | 91.3% |
| Ser | Carboxyl (=O) | A | 21 | 5 | 80.8% |
| His | Carboxyl (-OH) | A | 21 | 3 | 87.5% |
| Lys | Phosphate | A | 20 | 78 | 20.4% |

**Table S7. The precision and recall of the MAPSHB-Ligand model at various classification threshold values. The recommended classification threshold is shown in bold.**

| Classification Threshold | Precision | Recall |
|---|---|---|
| 0.996 | 98% | 56% |
| 0.979 | 93% | 68% |
| 0.943 | 89% | 75% |
| **0.870** | **86%** | **80%** |
| 0.807 | 85% | 81% |
| 0.740 | 84% | 83% |
| 0.656 | 82% | 85% |
| 0.555 | 79% | 87% |
| 0.448 | 77% | 92% |
| 0.304 | 74% | 94% |
| 0.159 | 70% | 95% |
| 0.062 | 67% | 96% |

**Table S8. The precision and recall of the gradient boosting-only model at various classification threshold values.**

| Classification Threshold | Precision | Recall |
|---|---|---|
| 0.996 | 100% | 38% |
| 0.979 | 99% | 57% |
| 0.943 | 94% | 66% |
| 0.870 | 88% | 72% |
| 0.807 | 87% | 74% |
| 0.740 | 86% | 76% |
| 0.656 | 86% | 79% |
| 0.555 | 83% | 81% |
| 0.448 | 83% | 83% |
| 0.304 | 81% | 86% |
| 0.159 | 79% | 89% |
| 0.062 | 75% | 92% |

**Table S9. PBD IDs of the 1070 protein-ligand complexes analyzed in this work.**

| | | | | | | | | | | | |
|---|---|---|---|---|---|---|---|---|---|---|---|
| 1V0K | 1V0L | 1V0M | 1VL9 | 1W23 | 1W3L | 1W8F | 1WUI | 1WUK | 1X8N | 1X8O | 1X8P |
| 1X8Q | 1XF6 | 1Y55 | 1Y93 | 1YFQ | 1YLJ | 1YLT | 1YQS | 1YWA | 1YWB | 1YWC | 1YWD |
| 1Z3N | 1Z8A | 1ZJY | 1ZJZ | 1ZK4 | 1ZL0 | 2ABA | 2ABB | 2ABS | 2AGT | 2AIB | 2AT0 |
| 2AT3 | 2AVO | 2AVS | 2AYW | 2BAX | 2BF6 | 2BHU | 2BOG | 2BOI | 2BT9 | 2BV4 | 2BW4 |
| 2BZZ | 2CHH | 2CI1 | 2CNQ | 2CZQ | 2D5M | 2EKT | 2EVW | 2EWI | 2EWK | 2EWU | 2F01 |
| 2FBA | 2FFY | 2FHL | 2FN3 | 2FVY | 2FZD | 2GH7 | 2GJ3 | 2GUD | 2HEU | 2HS1 | 2I16 |
| 2I17 | 2IDT | 2IDW | 2J8T | 2JDH | 2JDK | 2JHF | 2JJJ | 2NMY | 2NMZ | 2NRL | 2NRM |
| 2NUI | 2NX0 | 2O90 | 2OB3 | 2OF8 | 2OFR | 1DY5 | 2OSX | 2PEV | 2PF8 | 2PFH | 2PZN |
| 2QJ7 | 1A6K | 2QXW | 2R31 | 1A6M | 2V3I | 2VFR | 2VHA | 2VU7 | 2VXN | 2VZC | 2W14 |
| 2W15 | 2W39 | 2WBQ | 2WF7 | 2X9G | 2XA5 | 2XJP | 2XOD | 2XWV | 2Y62 | 2YYX | 2Z6W |
| 2ZD8 | 2ZQ9 | 2ZQA | 3A2O | 3A72 | 3AGN | 3AGO | 3AHW | 3AJ4 | 3AKS | 3AKT | 3AL7 |
| 3ALD | 3AYJ | 3BCJ | 3BVX | 3BWH | 3C76 | 3C77 | 3CNJ | 3D1P | 3D1X | 3D1Y | 3D20 |
| 1F9I | 3DJJ | 3DJK | 3DK1 | 3DK8 | 3E2O | 3E4G | 3E5T | 3E8M | 3EWW | 3EWY | 3F8V |
| 3FA0 | 3FX5 | 3G5S | 3GHR | 3GHS | 3GHT | 3HD2 | 3HGP | 1FSG | 3ILO | 3IP0 | 3IQU |
| 3JYO | 3L8W | 3LB2 | 3LBO | 3LEP | 3LL2 | 1G4I | 3LO8 | 3LZ3 | 3LZ5 | 3M0I | 3M4H |
| 3MWS | 3NBC | 3NDX | 3NON | 3NOQ | 3NOV | 3NU3 | 3NVS | 3NYC | 3O1C | 3O1X | 3O5R |
| 3ODV | 3ONC | 3P6D | 3P6E | 3P84 | 3P8J | 3PFZ | 3PYP | 3QGZ | 3QM5 | 3QM7 | 3QM8 |
| 3QM9 | 3QMA | 3QPA | 3QPC | 1GQV | 3RPE | 3RY1 | 3RY2 | 3SAX | 3SM8 | 3SNF | 1GVT |
| 1GVU | 1GVW | 3TU8 | 3U2C | 3U3H | 3U7Q | 1H11 | 3ULJ | 3VIF | 3VIG | 3VII | 3VIJ |
| 3VIK | 3VIM | 3VM9 | 3VUP | 1H5V | 3W07 | 3W5H | 3WA2 | 3WH7 | 3WMV | 3WOU | 3WVM |
| 3WYQ | 3X0K | 3X0O | 3X1X | 3X2G | 3X2H | 3X2L | 3X2M | 3X32 | 3X33 | 3X34 | 3X35 |
| 3ZIY | 3ZOJ | 3ZSJ | 4A29 | 4A7S | 4A7U | 4A7V | 4AWS | 4AWT | 4AYO | 4AYP | 4AYQ |

| | | | | | | | | | | | |
|---|---|---|---|---|---|---|---|---|---|---|---|
| 4AYR | 4B1M | 4BJ0 | 4BLI | 4BM8 | 4BPS | 4BS0 | 4BT3 | 4BT5 | 4BVM | 4CD5 | 4CE8 |
| 4CNG | 4CNN | 4CPE | 4CSS | 4CST | 4DRO | 4E9S | 4EA7 | 4EA8 | 4EA9 | 4EGU | 4EIE |
| 4EIF | 4EUZ | 4FCF | 4FEG | 4FH4 | 4GCA | 4GDA | 4GG1 | 4GM0 | 4GNU | 4GOG | 4H1A |
| 4HE9 | 4HL2 | 4HVU | 4ID0 | 4IGS | 4IZX | 4J5C | 4JED | 4KGD | 4KXU | 4KXV | 4KXW |
| 4KXX | 4LAU | 4LAZ | 4LB3 | 4LB4 | 4LBR | 4LBS | 1IXB | 4M5I | 4M5L | 4MIJ | 1J0P |
| 4MNC | 4MX6 | 4N0K | 4NFS | 4NG5 | 4NQR | 1JCJ | 1JCL | 4PF3 | 4PR4 | 4PRR | 4PRT |
| 4PSS | 4PST | 4PSY | 4PZH | 4Q68 | 4Q9W | 4QBX | 4QQS | 4QR6 | 4QRN | 4QXI | 1JK3 |
| 4RGC | 4RQR | 4RV5 | 4TJZ | 4TKB | 4TKH | 4TKJ | 4TWU | 4TWV | 4TX0 | 4TXR | 4U9I |
| 4UA9 | 4UDY | 4UHD | 4UHF | 4URF | 4UTJ | 4W7W | 4WJX | 4WK9 | 4WKA | 4WKH | 4WUI |
| 4WZ4 | 4X5P | 4XD2 | 4XOT | 4XZH | 4Y4J | 4Y5L | 4YCK | 4YS1 | 4YU1 | 4YXU | 4YYT |
| 4ZGF | 1KCC | 5A0Y | 5A1I | 5A8C | 5A92 | 1KDV | 1KDY | 5AKR | 5AN7 | 5B28 | 5B8D |
| 5BR4 | 1KJQ | 5CTV | 1KMS | 1KMV | 1KOI | 5DGJ | 5DGT | 5DOH | 5DP2 | 5DZE | 5EI4 |
| 5EKY | 5EL9 | 5ELB | 5ETK | 5ETO | 5F82 | 5FI3 | 1KWF | 5G18 | 1KZK | 5IDA | 5IDB |
| 5IG6 | 5IMC | 5J1N | 5JBX | 5JUG | 5KCP | 5KH9 | 5LVO | 5M03 | 5M17 | 5M3W | 5M5D |
| 5MEH | 8A3H | 1LQT | 1LUQ | 1M1Q | 1M1R | 1MNZ | 1N4U | 1N55 | 1N62 | 1N9B | 1NAZ |
| 1NH0 | 1NKI | 1NWZ | 1OCQ | 1OD3 | 1OEX | 1OH0 | 1ONG | 1OT9 | 1OTA | 1OTB | 1PMH |
| 1PQ8 | 1PWC | 1PWG | 1PWL | 1PWM | 1Q6Z | 1QV0 | 1QV1 | 1R2Q | 1S5M | 1S5N | 1SBY |
| 1SF5 | 1SXW | 1SXX | 1SXY | 1SY1 | 1SY2 | 1SY3 | 1T2D | 1T41 | 1TJ9 | 1TT8 | 1UFY |
| 1UNQ | 1US0 | 1C75 | 1UZV | 5B4J | 5GGB | 5GV7 | 5GV8 | 5HYV | 5J6Y | 5KMW | 5LJP |
| 5LSH | 5LUN | 5M28 | 5M2T | 5MH1 | 5MK9 | 5MTU | 5N8J | 5N8W | 5NCG | 5NE5 | 5NLD |
| 5NLH | 5NY6 | 5O2X | 5O99 | 5OF5 | 5OF6 | 5OGJ | 5OK2 | 5OLR | 5OU0 | 5OUJ | 5OUK |
| 5P9O | 5P9V | 5SW1 | 5T39 | 5TDB | 5VLE | 5WGI | 5X9L | 5X9M | 5XBX | 5XEC | 5XTL |
| 5XU2 | 5Y9Z | 5YOK | 5YYP | 5YYQ | 5YYR | 5Z3E | 5Z3F | 5ZIO | 5ZJ2 | 6BT6 | 6C17 |
| 6C1J | 6CNW | 6CW7 | 6D1I | 6EKZ | 6ENI | 6EV7 | 6EV8 | 6F7R | 6F81 | 6F82 | 6F84 |
| 6FIH | 6FK2 | 6FM7 | 6FMC | 6FPO | 6FTF | 6FWG | 6FWJ | 6FWP | 6G0V | 6G1G | 6G1I |
| 6G3Q | 6GDC | 6GH7 | 6GKE | 6GM9 | 6GX2 | 6H10 | 6H40 | 6H5G | 6HA3 | 6HAD | 6HAV |
| 6HMH | 6HMQ | 6HMT | 6HQX | 6HR3 | 6HSA | 6I03 | 6I0W | 6I1U | 6I3E | 6IJY | 6IXD |
| 6J5S | 6J64 | 6JGH | 6JGI | 6KBY | 6KKZ | 6KL0 | 6KL1 | 6M8F | 6M8W | 6M8Y | 6MAP |
| 6MM2 | 6MU0 | 6MZ1 | 6MZ2 | 6MZ3 | 6N3X | 6NNR | 6O91 | 6OA7 | 6OWM | 6P0Z | 6P2L |
| 6PWS | 6Q2Y | 6Q4D | 6Q4H | 6QLN | 6QLR | 6QLS | 6QLU | 6QW9 | 6QWA | 6QWB | 6R2Z |
| 6R34 | 6R3N | 6RHF | 6RK0 | 6S2M | 6S2S | 6SP6 | 6TD0 | 6UO3 | 6UQU | 1V0N | 1VB0 |
| 1VBW | 1VYR | 1W66 | 1W7J | 1CC8 | 1WY3 | 1XG0 | 1YRI | 1YS1 | 1ZWP | 2AGY | 2AOG |
| 2AT8 | 2AVM | 1CXQ | 1CZB | 2BWI | 2CE2 | 2FOS | 2FOU | 2GEW | 2GG2 | 2GG9 | 2GZ5 |
| 2H5O | 2HBW | 2HXS | 2I4A | 2IDU | 2J9J | 1DS1 | 2JE4 | 2NNO | 2NNS | 2NNV | 2O5G |
| 2O6N | 2OIZ | 2PVB | 2PVE | 2PWA | 2QXI | 2R0X | 2RH2 | 2RHF | 1EA7 | 2V1M | 2V89 |
| 2V8F | 2VB1 | 2VHK | 2VHR | 2VI2 | 2VI3 | 2VI4 | 2W72 | 2WEG | 2WFI | 2WFJ | 2WYT |
| 2X46 | 2X4K | 2XM4 | 2XOM | 2XTT | 2XU3 | 2YHG | 2YKZ | 2YL0 | 2YL1 | 2YL3 | 2YL7 |
| 2YLG | 2Z8A | 2ZPM | 1ETM | 1ETN | 2ZQC | 3A0M | 3AJN | 3AKQ | 3AKR | 3AL1 | 3B3R |
| 3BVA | 3C78 | 3CXZ | 3DHA | 3DK9 | 3E6Z | 3F17 | 3FIL | 3FMU | 3G46 | 3G9X | 3GMX |
| 3GYI | 3GYJ | 3HS4 | 3I06 | 3I94 | 3JU4 | 3KFH | 1G2Y | 3L5L | 3L5M | 3LZT | 3MCW |
| 1GA6 | 3NED | 3NOO | 3O4P | 3O89 | 1GKM | 3Q46 | 3Q8J | 1GMX | 3QL9 | 3QM6 | 3QZR |
| 3R2Q | 3R4G | 3R59 | 3RDA | 3RZN | 3S6E | 3SK2 | 3SU6 | 1GVV | 1GWE | 3TG2 | 3U8I |
| 3U97 | 3UAD | 3V7X | 1H4G | 3VBD | 3VN3 | 3VRC | 3WAR | 3WH1 | 3X0L | 3ZQV | 3ZQY |
| 3ZTM | 3ZTZ | 4AFF | 4AOH | 4AS8 | 4B4E | 4B5N | 4B5O | 4BLK | 4BLL | 4BM1 | 4C1H |
| 4C4R | 4CO3 | 4DRN | 4DRQ | 4EJD | 4EKF | 4FPT | 4FRC | 4FU5 | 4FVN | 4FVO | 4G9E |
| 4G9S | 4GJZ | 4GNR | 4GP9 | 4HBU | 4HE6 | 4HUA | 4HVW | 4J1P | 4J5E | 4JFI | 4JFJ |
| 4JFM | 4JP6 | 4JP7 | 4LQT | 4M51 | 4MKN | 4MZB | 4MZC | 4N1I | 4N9S | 4N9V | 4NSV |
| 4NSY | 4NV3 | 4NYX | 4O6Q | 4O6U | 4OMO | 4OOY | 4OP5 | 4PLZ | 4PNO | 1JFB | 1JFC |
| 4PZ3 | 4Q08 | 4Q2L | 4QB3 | 4QL3 | 4QMC | 4QYT | 4REK | 4RWC | 4U9H | 4UA7 | 4UAA |
| 4UTI | 4UTM | 4UYT | 4W7K | 4W7L | 4W9Q | 4WES | 4WPG | 4WPK | 4WS6 | 4WW6 | 4WX4 |
| 4X6H | 4X9Y | 4XWR | 4XXG | 4Y38 | 4Y45 | 4YAA | 4YEO | 4YX4 | 4YXI | 4YXO | 4ZA9 |
| 4ZC9 | 5A71 | 5ABO | 5AE0 | 5AGF | 5AOU | 5AUL | 5B27 | 5BT3 | 5CE4 | 5CTM | 5D66 |
| 5DRS | 5E1N | 5E7W | 5E9N | 5EKW | 5EMB | 1KT6 | 5ETP | 5FAF | 5FAH | 5FBF | 5FG6 |
| 5GJI | 5H5Q | 5HBS | 5I40 | 5I6L | 5I6M | 5I86 | 5I89 | 5IM9 | 5IMA | 5IMF | 5IMV |
| 5IMZ | 5INY | 5IQX | 5J0D | 5JGT | 5JH8 | 5KTW | 5LSV | 5MAE | 5MAJ | 5SVY | 5SY9 |
| 5SYA | 5TFQ | 5U4H | 5WS7 | 1LKK | 1LUG | 1M40 | 1MXT | 1N1P | 1N40 | 1N4V | 1N4W |
| 1NNF | 1O5X | 1OD8 | 1OT6 | 1P9G | 1PJX | 1PM1 | 1QXY | 1RG8 | 1RQW | 1RWY | 1S0R |
| 1BYZ | 1T8K | 1TJX | 1UOW | 1UOZ | 1UWC | 5HUB | 5I5B | 5JSY | 5LJQ | 5LJT | 5LL8 |
| 5LLC | 5M78 | 5MB5 | 5NGX | 5NKF | 5NKG | 5NVG | 5NXV | 5NXW | 5NY1 | 5O45 | 5OBK |
| 5OFS | 5OGN | 5OGO | 5OGP | 5ONK | 5OTN | 5OXC | 5P9J | 5QHU | 5QI2 | 5QI5 | 5QI7 |
| 5TIF | 5U3A | 5VNY | 5W0G | 5XP6 | 5XPS | 5XS6 | 5XSA | 5XSB | 5XSM | 5XT4 | 5XTV |

| | | | | | | | | | | | |
|---|---|---|---|---|---|---|---|---|---|---|---|
| 5XTX | 5XUF | 5XVT | 5YCE | 5ZGE | 5ZGI | 5ZH1 | 5ZJ1 | 5ZX8 | 6ADF | 6BG3 | 6BG5 |
| 6BQA | 6C1X | 6C79 | 6CHE | 6CNU | 6DBC | 6DCM | 6DDJ | 6DI1 | 6DYF | 6E1Z | 6ESM |
| 6EUW | 6EV6 | 6FGG | 6FIY | 6FJ6 | 6FNG | 6FNK | 6FO5 | 6G7N | 6HMB | 6HMC | 6HMD |
| 6HR0 | 6HRI | 6HS9 | 6I74 | 6I7Y | 6IC1 | 6IIP | 6JGJ | 6MYE | 6N59 | 6NJ3 | 6Q41 |
| 6Q48 | 6Q49 | 6Q4E | 6Q4G | 6Q4J | 6Q4K | 6Q5Q | 6Q7B | 6Q7C | 6Q7D | 6Q7E | 6Q7G |
| 6QAZ | 6QLP | 6QLQ | 6QU6 | 6QW8 | 6R1D | 6R33 | 6RO3 | 6RTP | 6RYN | 6S14 | 6S17 |
| 6S1H | 6S25 | | | | | | | | | | |

**Table S10. PBD chemical IDs of the small molecule ligands in the 1272 protein-ligand SHBs.**

| | | | | | | | | | | | |
|---|---|---|---|---|---|---|---|---|---|---|---|
| XYP | XDN | MPD | PLP | BGC | FUC | MRD | HEM | DBV | BTN | HAE | ACT |
| SUC | BSA | NDP | 3NA | NAP | NAI | NAD | TLA | FMN | ACP | CIT | FID |
| ERG | MK1 | ONO | SIA | MFU | MMA | ACY | MAN | BMA | KOR | AMZ | ADP |
| MES | 6HE | CAG | TRS | SM3 | FMT | TOL | FAD | 017 | LDT | MHO | 0QS |
| ROC | BTB | NEU | 393 | HC4 | XX6 | PGH | WR2 | ZZU | G7P | SLB | G3P |
| MER | CEO | PCZ | KNJ | AHR | 3AM | 2AM | SEP | WZ5 | FDE | G55 | G05 |
| NRQ | U1P | U5P | HED | BME | KNI | PE0 | FRW | PRP | APC | 4PO | 388 |
| ZST | LAT | RIT | CSO | NHE | 478 | S3P | GPJ | EPE | ADN | FK5 | ZGB |
| ZGC | CTT | MIR | BCN | FNK | 2ZS | STA | 0EM | SUZ | 03W | IMD | FCT |
| LGC | NOJ | CBI | GBH | SGC | SSG | TPQ | FCB | A2G | STE | AR6 | GLC |
| BOG | MLT | 5UD | ALE | LDP | SMD | MVL | IFL | FRU | XYS | GMK | A6J |
| 3EB | 6NT | WTZ | 23B | PLM | SAH | LUV | CWX | CWK | 0MD | JB2 | JB3 |
| JBT | 5GP | MEM | MA4 | PYR | 2X9 | TZD | IM2 | G52 | ZZ7 | ACE | GSF |
| 64I | 7I6 | GTS | TDP | W8X | 1WW | M15 | 1WX | 4O9 | 4O8 | MH2 | GTR |
| 173 | SIN | NAJ | PFB | HPD | HFN | 2W8 | 2W9 | 2WB | NAG | PIA | 30L |
| 37V | 1DF | I98 | COM | DKA | DAO | MYR | ZND | 384 | CB4 | 0NM | BUA |
| 1PS | 8CM | CBS | 3VU | 3XJ | NDG | RAM | 48I | 4JE | S2O | DYA | SAM |
| PPK | PHL | LLK | V64 | 5NV | 5DL | GAL | GLA | AZR | JE2 | 6B3 | MLI |
| COA | ATP | MNM | 7D1 | DGO | DMJ | IDC | FAE | PGA | MCN | KI2 | PPF |
| LOV | EQU | PNM | HE0 | BFI | CZH | GNP | XYL | OXL | ID5 | PHB | 4IP |
| BLA | 8GD | TPP | KTS | OGA | MLR | URI | BCD | DGL | 8TB | KIF | LBT |
| V90 | BG6 | AV5 | AW8 | AVT | 7JF | 7JM | MHA | DA2 | JSD | TSN | HEC |
| P23 | OCS | GSH | 8Z0 | 3GK | DNX | THG | IAC | 4LU | BYN | SOR | NXL |
| DUE | 7CI | E9K | EGZ | FO9 | EYW | FJ8 | FQZ | T6F | FE9 | GDQ | TPO |
| FLC | M4S | H1Z | 8DG | B0F | AP5 | B4P | GYS | CRO | JC4 | 5RP | KBD |
| CXM | HCQ | HHT | HGH | J5Q | J4N | HRK | J62 | MK7 | JQZ | JQH | KJK |
| 4D6 | QCP | PBC | | | | | | | | | |

## References


1. Wlodawer, A., Minor, W., Dauter, Z. & Jaskolski, M. Protein crystallography for non-crystallographers, or how to get the best (but not more) from published macromolecular structures. *FEBS J.* **275**, 1–21 (2008).
2. Li, W. & Godzik, A. Cd-hit: a fast program for clustering and comparing large sets of protein or nucleotide sequences. *Bioinformatics* **22**, 1658–1659 (2006).
3. Fu, L., Niu, B., Zhu, Z., Wu, S. & Li, W. CD-HIT: accelerated for clustering the next-generation sequencing data. *Bioinformatics* **28**, 3150–3152 (2012).
4. Case, D. *et al. AMBER 2016* (University of California, San Francisco, 2016).
5. Riley, P. Three pitfalls to avoid in machine learning. *Nature* **572**, 27–29 (2019).
6. Zhou, S., Liu, Y., Wang, S. & Wang, L. Effective prediction of short hydrogen bonds in proteins via machine learning method. *Sci. Rep.* **12**, 469 (2022).
7. Hastie, T., Tibshirani, R., Friedman, J. H. & Friedman, J. H. *The elements of statistical learning: data mining, inference, and prediction*, vol. 2 (Springer, 2009).
8. Friedman, J. H. Greedy function approximation: A gradient boosting machine. *Ann. Stat.* **29**, 1189–1232 (2001).
